# Human Performance in a Realistic Instrument-Control Task during Short-Term Microgravity

**DOI:** 10.1371/journal.pone.0128992

**Published:** 2015-06-17

**Authors:** Fabian Steinberg, Michael Kalicinski, Marc Dalecki, Otmar Bock

**Affiliations:** 1 Institute of Physiology and Anatomy, German Sport University, Cologne, Germany; 2 Institute of Sport Science, Johannes Gutenberg University, Mainz, Germany; 3 School of Kinesiology and Health Science, Centre for Vision Research, York University, Toronto, Canada; Charles P. Darby Children's Research Institute, UNITED STATES

## Abstract

Previous studies have documented the detrimental effects of microgravity on human sensorimotor skills. While that work dealt with simple, laboratory-type skills, we now evaluate the effects of microgravity on a complex, realistic instrument-control skill. Twelve participants controlled a simulated power plant during the short-term microgravity intervals of parabolic flight as well as during level flight. To this end they watched multiple displays, made strategic decisions and used multiple actuators to maximize their virtual earnings from the power plant. We quantified *control efficiency* as the participants’ net earnings (revenue minus expenses), *motor performance* as hand kinematics and dynamics, and *stress* as cortisol level, self-assessed mood and self-assessed workload. We found that compared to normal gravity, control efficiency substantially decreased in microgravity, hand velocity slowed down, and cortisol level and perceived physical strain increased, but other stress and motor scores didn’t change. Furthermore, control efficiency was not correlated with motor and stress scores. From this we conclude that realistic instrument control was degraded in short-term microgravity. This degradation can’t be explained by the motor and/or stress indicators under study, and microgravity affected motor performance differently in our complex, realistic skill than in the simple, laboratory-type skills of earlier studies.

## Introduction

The success of manned space missions depends critically on skilled human performance, such as spacecraft navigation and control of onboard scientific equipment. It therefore is disquieting that human skills were found to degrade in short- and long-term weightlessness: Pointing, tracking and grasping movements slowed down and/or became less accurate [1–7], and their cognitive demand increased [8–12]. Although the above work dealt with simple, laboratory-type skills, it has been suggested that astronauts’ regular daily activities are degraded as well [13]. However, such a generalization may be inadequate for two reasons. First, simple skills show different kinematics and dynamics when they are executed in a typical laboratory context versus in a more realistic context [14,15] and are differently affected by microgravity [16]. Second, simple skills may be differently affected by microgravity than complex daily routines. We therefore decided to evaluate the influence of short-term microgravity not on simple, laboratory-type skills but rather on a complex skill that bears a resemblance to astronauts’ instrument control routines.

The observed degradation of simple, laboratory-type skills in microgravity has been attributed not only to direct effects on the sensorimotor system [2,17–19], but also to indirect effects, mediated by stress [20,21]. Indeed, microgravity scenarios involve multiple stressors such as time pressure, ambient noise, lack of privacy, changed sleep pattern and imminent danger [9,22], and raise the levels of neurovegetative, endocrine and psychological stress markers during space missions [23,24], as well as during parabolic flights [25,26]. Since increased stress has been associated with impaired motor skills on Earth [27,28], especially when tasks are complex [29–31], it is quite conceivable that increased stress in microgravity contributes towards the impairment of complex skills in microgravity as well. We therefore decided to assess not only instrument control and basic motor performance of our participants, but also their level of stress.

Our working hypothesis was that instrument control is degraded in short-term microgravity, and that this deficit is mediated by motor impairments and/or by elevated stress.

## Material and Methods

### Participants

Twelve right-handed volunteers (7 males and 5 females) aged 28.8 ± 4.8 years participated. They had no prior experience in parabolic flight or sensorimotor research and no history of vestibular or sensorimotor deficits. Data were recorded during four parabolic flight campaigns held in Bordeaux (France) 2013 and 2014; each parabolic flight consisted of 30 intervals of 20 s duration under near-weightlessness (μG), each of them embedded in intervals of 20 s duration under increased weight (1.8G) and 30 intervals of 1–8 minutes duration under normal weight (1G). All participants underwent a clinical check prior to the study and received scopolamine (men received 0.7 mg and women 0.5 mg) approximately 1 hour before take-off to prevent motion sickness. The effects of Scopolamine start within an hour after intake and last for about eight hours [32]; since testing started about 90 minutes after intake and lasted for about four hours, we expected that possible effects of motion sickness and side effects of Scopolamine would influence subjects‘ performance evenly during the 1G and μG phases of our experiment. We nevertheless decided to guard against a differential influence by testing subjects in the order 1G - μG—μG- 1G (see below).

### Ethics statement

All volunteers were completely clarified about study procedure and aims and signed a written informed consent statement before participating in this study. The study involved human participants and was in accordance with the principles of the Declaration of Helsinki, which was pre-approved by the local Ethics Committee of the German Sport University and by the French Ethics Committee administered through the University of Caen.

### Setup

**Fig 1:**
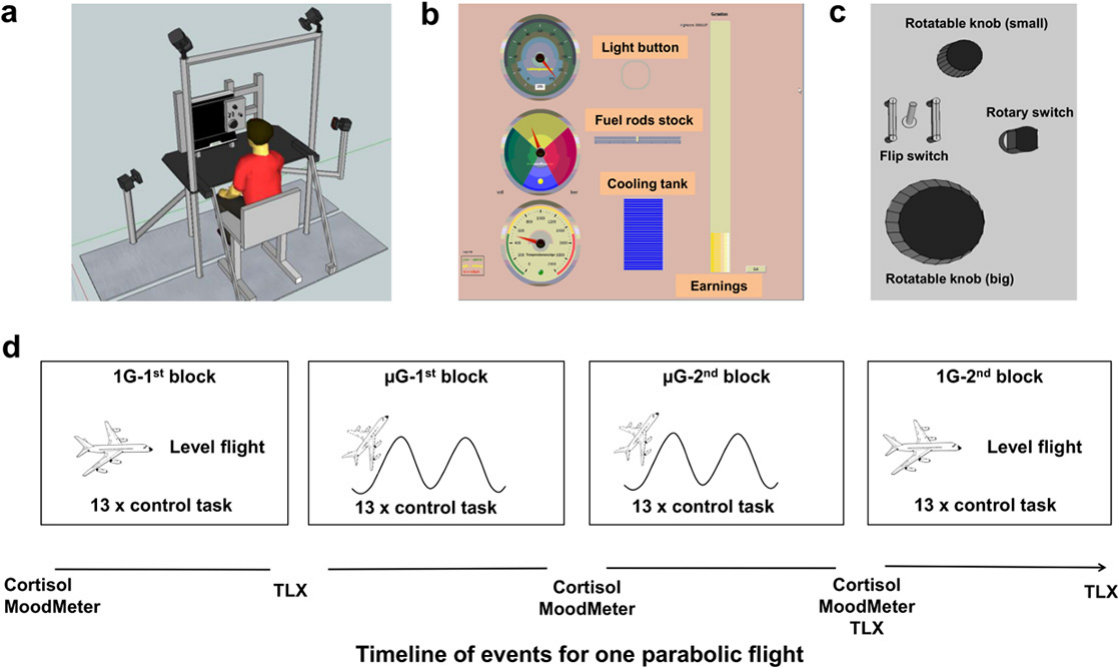
Experimental setup, control task and experimental timeline. **a:** Experimental setup for the use in parabolic flights. Shown is a participant sitting in a chair in front of the Eye tracker (incorporated in the screen) and the control panel within the metal frame, which serves as the construction for assembly into the parabolic flight plane. Four Bonita Vicon cameras for 3D hand motion capturing surround the participant. **b:** Screen of the simulated power plant with feedback displays regarding the requested power (top left), level of fuel rods (middle left), light button (top middle), temperature (bottom left), cooling tank (bottom middle) and earnings (right). The top left display element presents the inset for power requests. **c**: Enlargement of the control panel as shown in “a” with the small and big rotatable knobs, the rotary switch and the flip switch. The small rotatable knob controls the display element on the bottom left, the rotary switch the middle-left, the flip switch the light button and the big rotatable knob controls the top left element. **d:** Experimental time line for a participant during one flight day; shown are the points in time where the measurements were taken with respect to the flight profile along with the blocks of the control task. Cortisol stands for collection of saliva sample, the MoodMeter for mood assessment and the TLX for the NASA task load index.

Participants sat in front of a 17” screen with a built-in eye tracker system (Tobii T60, sampling rate: 60 Hz; Gaze data will be analyzed in a separate communication.) and were secured by a seatbelt in order to prevent free floating in μG. To the right of the screen was a control panel with four actuators (see Fig 1A, 1B and 1C): A cylindrical rotatable knob of 35 mm diameter, one of 70 mm diameter, a rotary switch of 17 x 25 mm size which could be turned in six steps of 20°, and a standard flip switch of 17 mm length. These actuators were selected for similarity with actuators aboard the International Space Station (see Fig 1C). Force sensors (ATI Nano 17) registered the grip forces at a sampling rate of 250 Hz and rotary encoders (RoHS RES20; 20Hz) registered the position of each actuator—except for the flip switch. Four Vicon Bonita cameras registered the positions of six infrared-light reflecting markers (6 mm in diameter), attached by double sided adhesive tape to the participants’ index fingertip, thumb and midpoint of the index finger’s metacarpal bone; the data were converted into 3D marker positions with a sampling rate of 240 Hz and an accuracy of 1 mm.

### Control task

Participants were engaged in a game that simulates a complex real-life scenario, the control of a nuclear power plant. To this end, seven displays were presented on the screen (see Fig 1B). A bar on the right showed the momentary earnings in Euros of the power plant; this was the most important display element, since the aim of the game was to maximize those earnings. A circular display at the top left indicated the momentary power production and–as an inset–the requested power production. Participants could adjust the momentary production by rotating the larger knob to reduce the difference between momentary and requested production, and thus to increase the rate of earnings. To make this task more challenging, requested production changed every 5 to 10 s such that participants permanently had to monitor and adjust power production. All participants were given the same sequence of power requests.

A circular display at the middle left indicated the momentary energy capacity of the fuel rod in use. This capacity decreased in proportion to the momentary power production and accordingly, a pointer rotated from the green to the red sector of the display. When it reached the red sector, participants had to insert a new rod such as to prevent a shutdown of the plant that would curtail their earnings. They inserted the rod by moving the rotary switch one step clockwise, which restored the pointer into the green sector and decremented the number of fuel rods in stock displayed in the center of the screen. After the last (sixth) rot was inserted, participants had to refill the stock by moving the rotary switch six steps counterclockwise. The refill incurred costs, i.e., the displayed earnings decreased.

A circular display at the bottom left showed the core temperature. The temperature increased proportionally with power production and, when the pointer reached the end of the red sector, the plant shut down. Thus to prevent a loss of earnings, participants had to monitor the core temperature and, when necessary, refill the cooling tank displayed at the bottom center of the screen. They did so by rotating the smaller knob.

Note that task complexity was augmented by the use of an incompatible actuator-display arrangement: the top knob controlled the bottom display, and vice versa. As another measure to increase task complexity, we simulated the night-day cycle by slowly darkening the screen over an interval of 11–20 s; by the end of that time, a beep and a light flash in the top middle of the screen prompted subjects to “turn on the lights’ by operating the flip switch. This happened once per episode, i.e., participants turned the light on by flipping the switch up in one episode, by flipping it down in the next, etc.

### Stress assessments

Subjective workload was assessed by a German translation of the NASA task load index (TLX), which consists of six items judged on a 20-point Likert scale. Mood was assessed by a modified version [25] of the Mood Meter [33], using 32 items rated on a 6-point Likert scale. Both questionnaires were administered in a paper-and-pencil form. To yield an objective measure of stress, saliva samples were taken to determine cortisol levels. This was done not only during the flight but also at the same times-of-day 24 hours later, to establish a baseline.

### Experimental protocol

One day before the flight, participants were familiarized with all procedures and practiced 13 episodes of the control task on the ground. Task difficulty (i.e. change of requested power scores) of training episodes was held at an equal level as in the experimental conditions; all participants reported to understand the task, which was additionally indicated by a positive earning (i.e. task success) and validated by subjective impression of investigators. On the day of flight, data were collected during level flight (1G) and during the near-weightlessness periods of parabolic flight (μG). The control task was subdivided into two blocks of 13 episodes of 20 s duration, such that each episode fitted into one μG interval. The first block was scheduled during 1G. All game settings and scores were then stored, the game was reset, and two further blocks were completed during μG. The stored game settings and scores were then re-instated, and a final block was given during 1G. Fig 1C summarizes this protocol along with the stress assessments.

### Data analysis

Kinematic and force data were reduced to a set of movement parameters by an interactive computer algorithm, as summarized under “motor performance” in Table 1. Signal processing filtering was performed by an eight-point simple moving average (SMA). Peak hand velocity (PHV) was calculated based on the velocity (cm/s) profile (in xyz-direction) for the marker attached to the wrist for each hand movement. The computer algorithm plotted the velocity profile and indicated PHV by a marker in order that each movement could be inspected visually for correctness and changed manually if necessary. Peak grip aperture (PGA) was calculated as the maximum 3D distance between the two markers that were attached to the thumbs´ and index fingers´ tops for each movement to a knob. For both parameters (PHV and PGA) visual markers were only set at a time interval of 1500 ms before contact to a knob to avoid inclusion of movements not directed to a knob. Maximum force (F) was calculated (via the force sensors) by the maximum knob compression in all (xyz-) force directions acting at the knob’s surface for each manipulation phase. Knob contact time (CT) was calculated as the time for the first force registration above baseline values until below or equal baseline values. Number of knob contacts ((KC) i.e. grasping movements) for each knob and across knobs were calculated for each episode and added up for each condition (1G, μG). Less than 5% of kinematic data were lost due to marker obtrusion. Parameter means across both 1G blocks and the means across both μG blocks were used for further analyses. As an index of movement variability the coefficients of variation (CV) for the parameters of motor performance (i.e. PHV, PGA, CT and F) were calculated. Control efficiency was determined as the sum of each net earnings (revenue minus expenses) of each 26 episodes of the respective condition (μG and 1G). Thus, this score represented the displayed earnings in Euros at the end of the second 1G block, as well as at the end of the second μG block. MoodMeter ratings were expressed as perceived physical strain (PEPS), psychological strain (PSYCHO) and motivational state (MOT), as per the test manual, while TLX ratings gave an overall workload score. Saliva Cortisol levels were expressed as difference between inflight and baseline values. All data were submitted to analyses of variance (ANOVAs) with repeated measures on Gravity (1G, μG) or on testing Time (early, middle, late) or on Gravity (1G, μG) crossed with Knob type (small, large, rot). Significant differences were explored with Bonferroni post-hoc tests.

**Table 1:** Summary and description of all test variables*.

Component	Abbreviation (unit)	Description
*Motor performance*		
Transport component of the hand	PHV	Peak hand velocity while grasping a knob
	PHVsmall (cm/s)	Peak hand velocity while grasping the small knob
	PHVlarge (cm/s)	Peak hand velocity while grasping the large knob
	PHVrot (cm/s)	Peak hand velocity while grasping the rotary switch
Grasp component of the hand	PGA	Peak Grip Aperture while grasping a knob
	PGAsmall (mm)	Peak Grip Aperture while grasping the small knob
	PGAlarge (mm)	Peak Grip Aperture while grasping the large knob
	PGArot (mm)	Peak Grip Aperture while grasping the rotary switch
Grip force	F	Peak grip force while grasping a knob
	Fsmall (N)	Peak grip force while grasping the small knob
	Flarge (N)	Peak grip force while grasping the large knob
	Frot (N)	Peak grip force while grasping the rotary switch
Grip time	CT	Knob contact time
	CTsmall (s)	CT for the small knob
	CTlarge (s)	CT for the large knob
	CTrot (s)	CT for the rotary switch
Number of grasping movements		
Knob contacts	KC	Count of knob contacts
	KCsmall (quantity)	Count of contacts of the small knob
	KClarge (quantity)	Count of contacts of the large knob
	KCrot (quantity)	Count of knob contacts of the rotary switch
*Stress*		
Mood Meter	PEPS	Mean of items for perceived physical strain
	PSYCHO	Mean of items for psychological strain
	MOT	Mean of items for motivational state
Saliva Cortisol	Cortisol (μg/dl)	Cortisol level on flight minus on baseline day
Task Load Index	TLX	Sum of all items
*Operational efficiency*		
Control efficiency	Efficiency (€)	Net final earnings in 1G and in μG

*Shown in the left column are all analyzed parameters, in the middle column the respective acronyms and units, and in the right column short parameter descriptions.

Additional detailed parameter descriptions and signal processing is described in “Data analysis”.

We also performed a stepwise multiple linear regression analysis, with the difference between 1G and μG scores of control efficiency as the dependent variable. As independent variables served the difference between 1G and μG scores of the motor parameters PHV, PGA, F and CT, and the difference between the first and last test scores of the Cortisol level, the TLX score and the MoodMeter scores for PEPS, PSYCHO and MOT. Effect sizes are reported only for significant results to avoid reporting negligible effect sizes. Effect sizes of ANOVAs were estimated as eta-squares (η^2^), where η^2^ > .01 indicates a small, η^2^ > .06 a medium and η^2^ >.14 a large effect [34]. Due to technical problems, some variables were only available from less than 12 participants, which is indicated in the respective analyses.

## Results

### Control task performance

**Fig 2:**
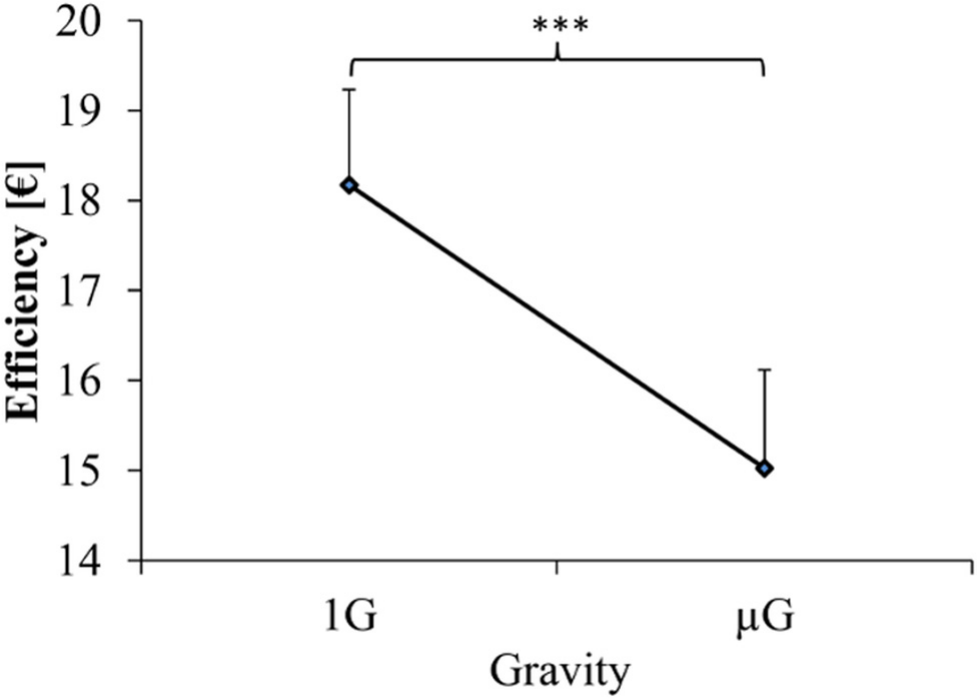
Control efficiency. Shown is the parameter control efficiency in normal (1G) and in microgravity (μG). 1G score is the total earned money across all 26 episodes of the control task performed in normal gravity; accordingly μG score represents the earnings of all 26 episodes of the control task in microgravity. Data are presented as means ± standard errors divided by 1000. *** = *p* < .001.

**Fig 3:**
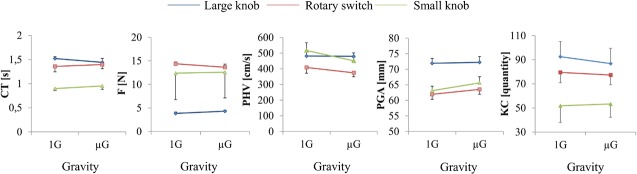
Motor performance. Shown are the interaction plots of grasping parameters subdivided by their values of the three knobs (large, rotary and small knob) in normal (1G) and in microgravity (μG). PHV represents peak hand velocity, PGA the peak grip aperture, CT the knob contact time, F the maximum force applied to the knobs and KC the number of grasping movements performed in each condition and for each knob. Mean KC values for all knobs were above 50, which were acceptable for calculating means of motor performance. For all parameters, significant effects were found between the large, rotary and small knob (all *p* < .001). Significant interaction (*p* < .05) emerged between Knob x Gravity for PHV with speed reduction of the small (*p* < .05) and rotary knob (*p* < .05) in microgravity, and no change for the big knob (*p* > .05). All other ANOVA factors were not significant (*p* > .05). Data are presented as means ± standard errors, additional statistics are summarized in Table 2.

As depicted in Fig 2, participants earned less money in μG compared to 1G. The difference between Gravity conditions (1G, μG) was statistically significant (*F* (1, 11) = 19.714, *p* < .001, η^2^ = .64) and it was substantial, amounting to 17.6% less earnings. Fig 3 illustrates all motor scores in μG and 1G, and Table 2 summarizes the pertinent ANOVA outcome. Gravity had no significant effect on any motor parameter, the factor Knob (large, rotary and small knob) expectedly had significant effects on all parameters (PHV, PGA, F, CT, KC), and the interaction term (Knob x Gravity) was significant only for PHV. The latter finding reflects, according to Fig 3, a reduction of hand velocity in μG for the small knob and the rotary switch (both *p* < .05), but not for the large knob (*p* > .05). The magnitude of this reduction averaged for the small knob 8.3% and for the rotary switch 4.8%. Movement variability was in an acceptable range for all parameters (between 0.13 und 0.67) and Gravity (1G, μG) had no significant effect on any variability parameter (ANOVAs for CVs of PHV, PGA, F and CT were all *p* > .05).

**Table 2:** ANOVA of motor parameters.

Parameters & ANOVA factors	*F*	*p*	η^2^
PHV			
Knob	*F* (2, 18) = 21.380	< .001	.704
Gravity	*F* (1, 9) = .991	> .05	
Knob X Gravity	*F* (2, 18) = 3.960	= .04	.306
PGA			
Knob	*F* (2, 18) = 37.572	< .001	.807
Gravity	*F* (1, 9) = .982	> .05	
Knob X Gravity	*F* (2, 18) = 1.056	> .05	
F			
Knob	*F* (2, 18) = 29.541	< .001	.766
Gravity	*F* (1, 9) = .006	> .05	
Knob X Gravity	*F* (2, 18) = 2.057	> .05	
CT			
Knob	*F* (2, 18) = 22.807	< .001	.717
Gravity	*F* (1, 9) = .021	> .05	
Knob X Gravity	*F* (2, 18) = 1.453	> .05	
KC			
Knob	*F* (2, 18) = 36.744	< .001	.803
Gravity	*F* (1, 9) = 1.68	> .05	
Knob X Gravity	*F* (2, 18) = 2.439	> .05	

### Stress assessment

**Fig 4:**
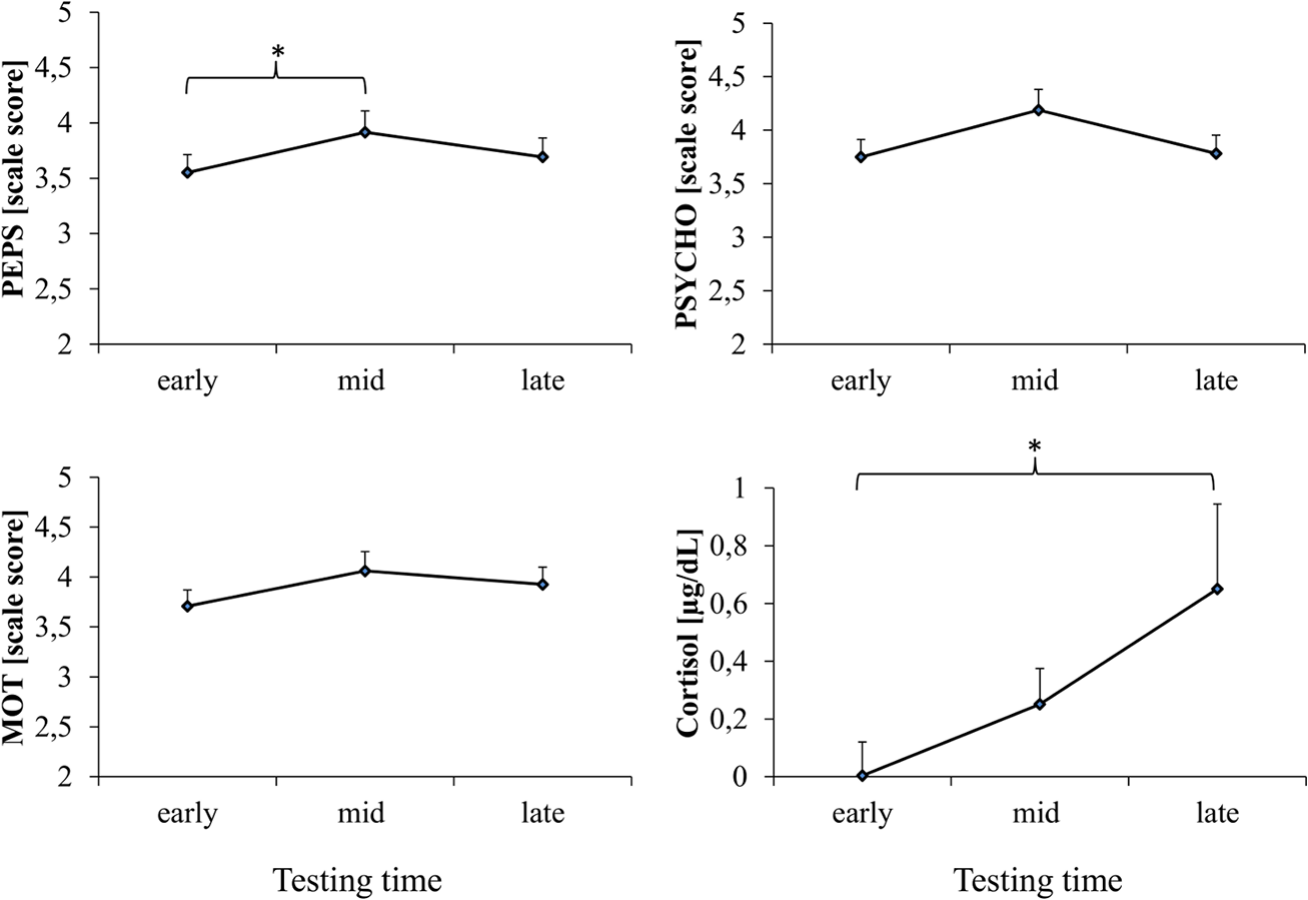
Stress indicators. Shown are the Cortisol levels and the three dimensions of the MoodMeter questionnaire. PEPS abbreviates the physical, PSYCHO the psychological and MOT the motivational mood dimension. One significant effect for the factor Time was found for PEPS (*p* < .05) between early and mid (*p* < .05); all other ANOVA factors were not significant (*p* > .05). Abbreviations of the x-axis indicate points in time corresponding to the flight profile, i.e. ‘early’ is taken before the 1G-1^st^ block, ‘mid’ is after the μG-1^st^ block and ‘late’ is before the 1G-2^nd^ block (cf. Fig 1D). Data are presented as means ± standard errors.

Separate ANOVAs for three dimensions of mood assessment yielded a significant effect of Time (early, mid, late) for PEPS (*F* (2, 22) = 4.525, *p* = .02, η^2^ = .29), but not for PSYCHO and MOT (both *p* > .05). Post-hoc analysis of PEPS revealed significant differences only between early and middle (*p* = .02): only the middle phase of μG (i.e. after 15 parabolas including performance of 13 control task episodes) was considered to be physically more stressing, as illustrated in Fig 4. There was no significant effect of Time for TLX (*F* (2, 22) = .228, *p* > .05) but there was one for Cortisol (*F* (2, 20) = 4.093, *p* = .03, η^2^ = .29). Fig 4 illustrates a continuous increase of Cortisol levels from early to middle to late time, but post-hoc decomposition revealed a significant difference only between early and late (*p* < .05).

Stepwise multiple linear regression with an inclusion criteria for each variable was set at F values = 1 or higher led to the inclusion of only two parameters, PHV and Cortisol, and didn't reach statistical significance (*F* (2, 7) = 2.26; R^2^ = .39, *p* > .05, see Table 3)."

**Table 3:** Multiple stepwise regression of motor and stress changes on control efficiency change.

	*b*	*t*	*p*
PHV	-12.07	-1.85	> .05
Cortisol	-882.29	-1.32	> .05

## Discussion

The present study evaluated how human performance in a complex, realistic control task is affected by short-term microgravity, and whether changes of performance are associated with changes of basic motor functions and/or stress. We found no appreciable effects of microgravity on grasping kinematics and dynamics, except for reduced hand movement speed towards the smaller two actuators. This is in contrast to a variety of previous studies that observed an influence of μG on several grasping parameters during a simple laboratory-type task [5,11,7] as well as during a simple, everyday-like task [16]. From this we conclude that grasping is not uniformly affected by μG: Elementary grasping acts are influenced more than grasping movements embedded in a realistic instrument control skill.

We have observed before that μG may affect the kinematics of one task but not those of another task, and attributed this discrepancy to a differential allocation of the brain’s computational resources [11]. A similar interpretation may hold for the discrepant data on grasping: Our control task was quite captivating, which possibly enticed participants to allocate extra resources to the motor system and thus keep motor responses unchanged in μG; the simple grasping acts of previous studies were possibly less effective in increasing the resource supply. This interpretation is in accordance with our stress data. Perceived physical strain and Cortisol (an indicator of psychological [35] as well as physical stress [36]) increased in μG while psychological strain, motivational state and task load were not affected; this pattern of findings has been reported before [25], and it is consistent with the notion that physical but not psychological stress is elevated during parabolic flight as more resources are allocated to the motor system.

Besides the above effects of μG on grasping and stress, we also observed a substantial degradation of control efficiency. Similarly, previous work has reported a degradation of surgical skills in μG: the number of completed tasks [13] and their quality decreased [37]. The large drop of control efficiency is difficult to explain by the relatively subtle changes of grasping performance; however, it could well be related to the change of physical stress. In particular, the allocation of additional resources to motor control may have led to a shortage of resources for other task components, such as display monitoring and decision making. If so, changes of control efficiency in μG should be correlated with changes of stress, and less so with changes of grasping. However, multiple regression analysis revealed no substantial associations between the gravity-related changes of control efficiency, motor performance and stress. Thus, although stress influences the performance of complex motor tasks on Earth [29–31], we found no evidence that it mediates the change of control efficiency in microgravity.

This is not to say that control efficiency in μG is completely immune to stress. Stressors such as imminent danger and lack of privacy exist on parabolic flights, but have not been assessed in our study. Likewise, stress indicators such as α-Amylase, Prolactin and Epinephrine are known to influence cognitive and motor functions [25,28], but have not been registered in the present work. The possibility therefore remains that realistic working performance in μG is degraded because of aspects of stress that were not considered in our study. Likewise, it may be degraded because of motor deficits not captured in the present study. Alternatively, the degradation might reflect the effects of μG on mental functions such as affect, distributed attention, concentration, multitasking or decision-making.

Our finding, that the performance of a realistic control task is degraded in short-term microgravity should be extended by research during long-term space missions; if it persists, it would have practical implications for the planning of space activities and for the preflight training of astronauts.

## Supporting Information

S1 File Complete data of the study. (SAV)
